# The effects of Y-27632 on pial microvessels during global brain ischemia and reperfusion in rabbits

**DOI:** 10.1186/s12871-017-0331-5

**Published:** 2017-03-07

**Authors:** Noriyuki Shintani, Tadahiko Ishiyama, Masakazu Kotoda, Nobumasa Asano, Daniel I. Sessler, Takashi Matsukawa

**Affiliations:** 1 Surgical Center, University of Yamanashi Hospital, 1110 Shimokato, Chuo, 409-3898 Yamanashi Japan; 2 Department of Anesthesiology, Faculty of Medicine, University of Yamanashi, 1110 Shimokato, Chuo, 409-3898 Yamanashi Japan; 3 Department of Outcomes Research, Cleveland Clinic, Anesthesiology Institute, Cleveland, OH USA

**Keywords:** Y-27632, Global brain ischemia-reperfusion, Cranial window

## Abstract

**Background:**

Global brain ischemia-reperfusion during propofol anesthesia provokes persistent cerebral pial constriction. Constriction is likely mediated by Rho-kinase. Cerebral vasoconstriction possibly exacerbates ischemic brain injury. Because Y-27632 is a potent Rho-kinase inhibitor, it should be necessary to evaluate its effects on cerebral pial vessels during ischemia—reperfusion period. We therefore tested the hypotheses that Y-27632 dilates cerebral pial arterioles after the ischemia-reperfusion injury, and evaluated the time-course of cerebral pial arteriolar status after the ischemia-reperfusion.

**Methods:**

Japanese white rabbits were anesthetized with propofol, and a closed cranial window inserted over the left hemisphere. Global brain ischemia was produced by clamping the brachiocephalic, left common carotid, and left subclavian arteries for 15 min. Rabbits were assigned to cranial window perfusion with: (1) artificial cerebrospinal fluid (Control group, *n* = 7); (2) topical infusion of Y-27632 10^−6^ mol · L^−1^ for 30 min before the initiation of global brain ischemia (Pre group, *n* = 7); (3) topical infusion of Y-27632 10^−6^ mol · L^−1^ starting 30 min before ischemia and continuing throughout the study period (Continuous group, *n* = 7); and, (4) topical infusion of Y-27632 10^−6^ mol · L^−1^ starting 10 min after the ischemia and continuing until the end of the study (Post group, *n* = 7). Cerebral pial arterial and venule diameters were recorded 30 min before ischemia, just before arterial clamping, 10 min after clamping, and 5, 10, 20, 40, 60, 80, 100, and 120 min after unclamping.

**Results:**

Mean arterial blood pressure and blood glucose concentration increased significantly after global brain ischemia except in the Continuous group. In the Pre and Continuous groups, topical application of Y-27632 produced dilation of large (mean 18–19%) and small (mean; 25–29%) pial arteries, without apparent effect on venules. Compared with the Control and Pre groups, arterioles were significantly dilated during the reperfusion period in the Continuous and Post groups (mean at 120 min: 5–8% in large arterioles and 11–12% in small arterioles).

**Conclusions:**

Y-27632 dilated cerebral pial arterioles during reperfusion. Y-27632 may enhance recovery from ischemia by preventing arteriolar vasoconstriction during reperfusion.

## Background

Rho-kinase moderates many cellular functions including adhesion, morphology, proliferation and motility, and also smooth muscle contraction [1, 2]. A key step in smooth muscle contraction is phosphorylation of myosin light chain (MLC) by MLC kinase which is activated by Ca^2+^-calmodulin complex. MLC is dephosphorylated by MLC phosphatase. Rho-kinase enhances MLC phosphorylation and mediates vascular contraction [3]. Rho-kinase inhibitors thus attenuate MLC phosphorylation and inhibit smooth muscle contractility. Fasudil is a clinically available Rho-kinase inhibiter that reportedly increases cerebral blood flow [4]. Y-27632, another Rho-kinase inhibiter, dilates normal basilar arterioles [5] and cerebral pial arterioles [6].

Propofol is a commonly used anesthetic drug. Surgeries such as temporary arterial clipping during cerebral aneurysm surgery, carotid endarterectomy, repair of aortic arch aneurysm, unexpected cardiac arrest, etc. are clinical scenarios in which brain ischemia and reperfusion could occur under propofol anesthesia. Global brain ischemia-reperfusion during propofol anesthesia provokes persistent cerebral pial constriction [7]. Cerebral vasoconstriction possibly exacerbates ischemic brain injury [8]. Rho-kinase is activated in endothelial cells during cerebral ischemia and apparently contributes to consequent microcirculatory disturbances [9]. Fasudil, a Rho-kinase inhibiter, prevents cerebral vasospasm and reduces blood viscosity, and has been used therapeutically in patients with subarachnoid hemorrhage [10]. In addition, vasodilation after brain ischemia may provide sufficient oxygen and glucose and may remove acidic metabolites from the ischemic brain tissue for preservation of normal neuronal function [11]. Because cerebral vasodilation may help maintain brain perfusion during the vasoconstrictive phase of ischemia-reperfusion injury, it is important to evaluate the vasodilatory effect of Rho-kinase inhibitors during the global brain ischemia and reperfusion period.

We investigated the effect of Y-27632, a Rho-kinase inhibitor, on the cerebral pial arterial diameter changes during brain ischemia-reperfusion period using cranial windows in rabbits. Specifically, we evaluated the effects of application period of Y-27632 (before the ischemia, continuously during the study period, and after the ischemia) on ischemia-induced pial arteriolar diameter changes. Our hypotheses were that: 1) Y-27632 dilates cerebral pial arterioles during ischemia and reperfusion; 2) continuously applied Y-27632 produces maximum cerebral pial arteriolar dilation; and 3) Y-27632 restricted to the reperfusion period produces less cerebral pial arteriole vasodilation than continuous application throughout ischemia and reperfusion.

## Methods

This study was approved by the Committee on Animal Research at the University of Yamanashi, Japan. Experiments were performed on 28 Japanese white rabbits weighing 3.2–3.8 kg. After obtaining IV access in an ear vein, the animals were anesthetized with propofol (8 mg∙kg^−1^ IV bolus followed by an infusion of 36 mg∙kg^−1^∙h^−1^) [12]. A tracheostomy was performed and their lungs mechanically ventilated with an oxygen-air mixture.

End-tidal carbon dioxide tension (ETCO_2_) was continuously monitored (Vamos, Dräger medical, Tokyo, Japan). Based on ETCO_2_ measurements, the tidal volume and respiratory rate were adjusted to maintain arterial carbon dioxide tension (PaCO_2_) between 35 and 45 mmHg. A femoral-artery catheter allowed continuous monitoring of mean arterial blood pressure (MAP) and blood sampling. Bicarbonated Ringer’s solution (Bicarbon, Ajinomoto, Tokyo, Japan) was infused at 10 mL∙kg^−1^∙h^−1^.

After exposing the aortic arch, the brachiocephalic, left common carotid, and left subclavian arteries were isolated, and the rabbits were placed in the sphinx posture. A closed cranial window was implanted over the parietal cortex. With the scalp retracted, an 8-mm-diameter hole was made in the parietal bone. The dura and arachnoid membranes were cut; a ring with thin glass was then positioned over the hole and secured with bone wax and dental acrylic. The space under the window was filled with artificial cerebrospinal fluid (aCSF), which was composed with Na^+^ 151 mEq∙L^−1^, K^+^ 3.5 mEq∙L^−1^, Ca2^+^ 2.5 mEq∙L^−1^, Mg^2+^ 1.3 mEq∙L^−1^, HCO_3_^−^ 25 mEq∙L^−1^, urea 40 mg∙dL^−1^, and glucose 65 mg∙dL^−1^.

Three polyethylene catheters were inserted into the ring. One catheter was attached to a reservoir bottle containing aCSF which was continuously bubbled with 5% CO_2_ in air. The aCSF was suffused at 0.1 mL∙min^−1^. Other catheters served as an outlet for aCSF, and the third port measured pressure in the window. The level of the outlet was maintained approximately 5–6 cm above the window, thus the intracranial pressure was maintained at 5–6 cm H_2_O. The volume of fluid below the window was between 0.5 and 0.7 mL. Global brain ischemia was produced by simultaneously clamping the brachiocephalic, left common carotid, and left subclavian arteries for 15 min. It has been reported that, in gerbils, during the global ischemia-reperfusion period, there is a short phase of vasodilation followed by a prolonged phase of vasoconstriction [13]. We previously reported that pial arterioles were dilated temporarily and were constricted thereafter in the 15 min of global cerebral ischemia in rabbits [7]. Therefore, we employed 15 min of global brain ischemia.

Twenty-eight rabbits were assigned randomly to one of four groups with 7 rabbits per group. A sample-size estimate analysis indicated that 7 rabbits per group was sufficient to detect a 15% change in pial arteriolar diameter from the control values with a power of 0.8 and α < 0.05. In the Control group, artificial cerebrospinal fluid was infused into the cranial windows. In the Pre group, Y-27632 at 10^−6^ mol∙L^−1^ was infused from 30 min before the initiation of global brain ischemia for 30 min. Then artificial cerebrospinal fluid was infused during the ischemia-reperfusion period. In the Continuous group, Y-27632 at 10^−6^ mol∙L^−1^ was infused from 30 min before the global brain ischemia to 120 min after unclamping. In the Post group, Y-27632 at 10^−6^ mol∙L^−1^ was infused from 10 min after the global brain ischemia started until 120 min after unclamping. In our previous study, Y-27632 at 10^−6^ mol∙L^−1^ produced substantial cerebral pial arteriolar dilation [6]. On the basis of this dose-ranging study, we thus selected 10^−6^ mol∙L^−1^ as the concentration of Y-27632 for the current study.

Measurements of cerebral pial diameters were made at the following time-points: 30 min before global brain ischemia (baseline), just before global brain ischemia, 10 min after global brain ischemia, and at 5, 10, 20, 40, 60, 80, 100, 120 min after reperfusion. The diameters of 3–4 pial microvessels as available within each window were measured in each cranial window. Images of pial vessels were captured and recorded on a personal computer attached to a microscope (VH-5000, Keyence, Osaka, Japan) via video capture unit (VH-E500, Keyence, Osaka, Japan). The microscopic magnification was × 150. The resolution of the video system was 1600 × 1200 pixels. Diameters of pial vessels were measured off line using a digital video analyzer (VH Analyzer VH-H1A5, Keyence, Osaka, Japan) on a personal computer. The sizes of arterioles and venules were divided on the basis of initial diameters into 2 subgroups, > 70 (large), and ≤ 70 (small) micrometers. After the completion of the measurements, animals were euthanized by injection of potassium chloride in deep anesthesia with propofol.

### Statistical analysis

Results are presented as means ± SDs. Within-group comparisons in MAP, HR, body temperature, arterial blood pH, PaCO_2_, PaO_2_, and plasma concentrations of Na^+^, K^+^, glucose, and lactate in each experimental group were performed using repeated measures analysis of variance (ANOVA) and Dunnett’s *post hoc* testing. Cerebral pial vascular percent changes in diameter from the baseline in each group were examined via repeated measures ANOVA and Dunnett’s *post hoc* testing. Among-group comparisons were performed using factorial ANOVA and Newman Keuls *post hoc* testing. A *P* value less than 0.05 was considered statistically significant.

Power analysis indicated that a sample size of 7 rabbits per group was sufficient to detect a 15% change in pial arteriolar diameter from the control values with a power of 0.8 and α < 0.05. The primary outcome was average dilation of cerebral pial arterioles in response to Y-27632 during ischemia (Control and Post groups versus Pre and Continuous groups) and reperfusion (Control and Pre groups versus Continuous and Post groups). The secondary outcome was average pial arteriolar dilation over time depending on the timing of initiation of Y-27632 administration.

## Results

Mean arterial blood pressure (MAP) increased significantly, by about 27–39 mmHg, after clamping the brachiocephalic artery, left common carotid artery, and left subclavian artery in the Control, the Pre, and the Post groups. In contrast, heart rate (HR) remained largely unchanged in all groups. After unclamping, MAP, HR, and base excess decreased. Plasma glucose increased significantly in the Control, the Pre, and the Post groups (Tables 1, 2, 3 and 4). Physiologic variables were not different among the four groups.

**Table 1:** Physiologic measurements in the control group

	MAP	HR	pH	PaCO2	PaO2	Base excess	Na	K	Glucose
	(mm Hg)	(bpm)		(mm Hg)	(mm Hg)	(mEq/L)	(mEq/L)	(mEq/L)	(mg/dL)
baseline	107 ± 13	281 ± 47	7.36 ± 0.04	42 ± 5	199 ± 26	−2.0 ± 3.0	138 ± 3	3.7 ± 0.3	133 ± 19
clamp 10 min	144 ± 15*	269 ± 66	7.33 ± 0.06	42 ± 6	199 ± 17	−4.0 ± 1.7	136 ± 6	4.1 ± 0.9	212 ± 48*
declamp5min	98 ± 21	259 ± 59	7.32 ± 0.08	43 ± 7	204 ± 22	−4.3 ± 1.8	137 ± 4	3.7 ± 0.4	205 ± 29*
declamp10min	103 ± 9	272 ± 46	7.31 ± 0.07	44 ± 6	204 ± 22	−4.6 ± 2.2	136 ± 6	3.7 ± 0.4	211 ± 37*
declamp 20 min	104 ± 8	271 ± 38	7.31 ± 0.08	44 ± 7	194 ± 38	−4.3 ± 2.5	137 ± 3	3.8 ± 0.4	171 ± 52
declamp 40 min	98 ± 15	275 ± 38	7.34 ± 0.06	43 ± 7	198 ± 43	−2.9 ± 1.9	137 ± 3	3.8 ± 0.3	164 ± 47
declamp 60 min	103 ± 10	276 ± 67	7.35 ± 0.05	42 ± 6	203 ± 38	−2.6 ± 1.8	136 ± 5	3.9 ± 0.5	148 ± 35
declamp 80 min	103 ± 12	280 ± 32	7.37 ± 0.04	39 ± 5	202 ± 39	−2.6 ± 2.3	136 ± 7	4.1 ± 0.8	140 ± 30
declamp 100 min	104 ± 11	284 ± 32	7.36 ± 0.04	41 ± 5	196 ± 48	−2.3 ± 1.8	136 ± 6	4.1 ± 0.6	146 ± 30
declamp 120 min	106 ± 14	279 ± 40	7.36 ± 0.05	40 ± 6	193 ± 46	−3.0 ± 2.8	135 ± 6	4.1 ± 1.0	147 ± 40

Values are mean ± SD

*MAP* mean arterial blood pressure, *HR* heart rate, *baseline* before ischemia

* *P* < 0.05 compared with the values at baseline

**Table 2:** Physiologic measurements in the pre group

	MAP	HR	pH	PaCO2	PaO2	Base excess	Na	K	Glucose
	(mm Hg)	(bpm)		(mm Hg)	(mm Hg)	(mEq/L)	(mEq/L)	(mEq/L)	(mg/dL)
baseline	112 ± 15	266 ± 31	7.38 ± 0.03	41 ± 3	214 ± 50	−1.2 ± 2.8	139 ± 1	3.4 ± 0.4	144 ± 15
clamp 10 min	139 ± 25*	258 ± 39	7.31 ± 0.04	44 ± 4	193 ± 29	−4.1 ± 2.6	138 ± 2	3.8 ± 0.2	207 ± 55*
declamp5min	87 ± 10	236 ± 29	7.29 ± 0.04	46 ± 4	200 ± 28	−4.6 ± 1.9	138 ± 2	3.6 ± 0.3	228 ± 70*
declamp10min	95 ± 16	230 ± 27	7.29 ± 0.03	44 ± 4	201 ± 28	−5.5 ± 2.8	138 ± 2	3.6 ± 0.4	217 ± 64*
declamp 20 min	102 ± 20	235 ± 16	7.30 ± 0.03	43 ± 4	202 ± 26	−5.3 ± 2.3	137 ± 1	3.7 ± 0.4	209 ± 64*
declamp 40 min	105 ± 16	239 ± 22	7.32 ± 0.03	41 ± 4	206 ± 24	−5.0 ± 2.7	136 ± 2	3.8 ± 0.6	197 ± 73
declamp 60 min	105 ± 14	241 ± 28	7.33 ± 0.03	41 ± 5	206 ± 27	−4.5 ± 3.1	136 ± 2	4.0 ± 0.8	184 ± 73
declamp 80 min	101 ± 15	238 ± 19	7.33 ± 0.04	41 ± 3	204 ± 25	−4.3 ± 2.9	135 ± 2	4.1 ± 1.1	186 ± 67
declamp 100 min	104 ± 14	235 ± 23	7.33 ± 0.04	41 ± 3	187 ± 25	−4.2 ± 2.7	135 ± 3	4.2 ± 1.3	185 ± 66
declamp 120 min	104 ± 10	235 ± 33	7.32 ± 0.04	42 ± 2	183 ± 34	−4.2 ± 2.6	135 ± 3	4.3 ± 1.4	186 ± 62

Values are mean ± SD

*MAP* mean arterial blood pressure, *HR* heart rate, *baseline* before ischemia

* *P* < 0.05 compared with the values at baseline

**Table 3:** Physiologic measurements in the continuous group

	MAP	HR	pH	PaCO2	PaO2	Base excess	Na	K	Glucose
	(mm Hg)	(bpm)		(mm Hg)	(mm Hg)	(mEq/L)	(mEq/L)	(mEq/L)	(mg/dL)
baseline	110 ± 7	253 ± 44	7.40 ± 0.05	40 ± 3	189 ± 66	0.7 ± 2.7	137 ± 2	3.6 ± 0.3	139 ± 17
clamp 10 min	110 ± 46	229 ± 32	7.37 ± 0.06	43 ± 5	154 ± 75	−0.6 ± 2.5	138 ± 2	3.9 ± 0.4	163 ± 44
declamp5min	78 ± 36	213 ± 35	7.34 ± 0.08	45 ± 7	163 ± 63	−1.8 ± 3.0	137 ± 2	3.8 ± 0.5	159 ± 49
declamp10min	77 ± 27	215 ± 30	7.33 ± 0.08	46 ± 11	155 ± 66	−1.6 ± 3.3	138 ± 2	3.8 ± 0.5	160 ± 45
declamp 20 min	79 ± 26	221 ± 22	7.33 ± 0.09	46 ± 14	148 ± 57	−2.4 ± 2.6	137 ± 2	4.0 ± 0.6	156 ± 42
declamp 40 min	88 ± 26	221 ± 27	7.36 ± 0.07	43 ± 10	169 ± 64	−2.0 ± 2.3	137 ± 3	4.2 ± 0.8	151 ± 38
declamp 60 min	90 ± 28	218 ± 20	7.37 ± 0.05	42 ± 6	170 ± 65	−1.2 ± 2.1	136 ± 2	4.4 ± 1.2	146 ± 31
declamp 80 min	86 ± 28	211 ± 28	7.38 ± 0.06	41 ± 5	168 ± 63	−1.2 ± 2.3	136 ± 2	4.5 ± 1.3	147 ± 37
declamp 100 min	90 ± 29	212 ± 32	7.37 ± 0.05	41 ± 4	168 ± 62	−1.5 ± 2.4	136 ± 3	4.6 ± 1.4	147 ± 42
declamp 120 min	87 ± 27	208 ± 31	7.38 ± 0.06	40 ± 4	162 ± 75	−1.8 ± 2.8	135 ± 2	4.6 ± 1.4	151 ± 45

Values are mean ± SD

*MAP* mean arterial blood pressure, *HR* heart rate, *baseline* before ischemia

**Table 4:** Physiologic measurements in the post group

	MAP	HR	pH	PaCO2	PaO2	Base excess	Na	K	Glucose
	(mm Hg)	(bpm)		(mm Hg)	(mm Hg)	(mEq/L)	(mEq/L)	(mEq/L)	(mg/dL)
baseline	105 ± 8	252 ± 52	7.39 ± 0.04	42 ± 3	222 ± 43	0.6 ± 2.6	136 ± 2	3.7 ± 0.4	130 ± 24
clamp 10 min	144 ± 21*	255 ± 74	7.35 ± 0.06	42 ± 2	188 ± 40	−2.4 ± 4	135 ± 2	4.3 ± 1.0	188 ± 42*
declamp5min	99 ± 11	239 ± 64	7.31 ± 0.06	45 ± 3	203 ± 36	−3.0 ± 3.8	134 ± 2	3.9 ± 0.9	192 ± 47*
declamp10min	101 ± 12	248 ± 46	7.32 ± 0.07	44 ± 3	207 ± 36	−3.3 ± 3.9	135 ± 2	3.9 ± 1.0	195 ± 51*
declamp 20 min	102 ± 9	252 ± 42	7.34 ± 0.07	43 ± 3	210 ± 29	−2.7 ± 4.0	134 ± 3	4.0 ± 1.1	189 ± 56*
declamp 40 min	97 ± 12	256 ± 57	7.36 ± 0.06	42 ± 4	216 ± 24	−1.6 ± 3.8	133 ± 3	4.1 ± 1.2	172 ± 56
declamp 60 min	93 ± 4	255 ± 50	7.38 ± 0.07	41 ± 5	211 ± 28	−0.9 ± 3.6	134 ± 4	4.2 ± 1.2	152 ± 46
declamp 80 min	92 ± 18	233 ± 39	7.37 ± 0.06	40 ± 3	221 ± 25	−2.0 ± 2.1	133 ± 4	4.4 ± 1.1	147 ± 33
declamp 100 min	93 ± 16	257 ± 40	7.40 ± 0.07	39 ± 3	221 ± 28	−0.1 ± 3.8	133 ± 3	4.3 ± 1.2	140 ± 22
declamp 120 min	91 ± 10	257 ± 45	7.40 ± 0.06	39 ± 2	224 ± 32	−0.4 ± 3.5	132 ± 3	4.2 ± 1.1	136 ± 17

Values are mean ± SD

*MAP* mean arterial blood pressure, *HR* heart rate, *baseline* before ischemia

* *P* < 0.05 compared with the values at baseline

**Fig. 1:**
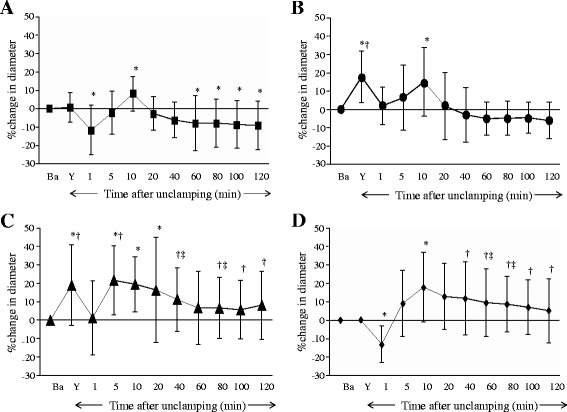
Percent changes in diameter in large (>70 μm) cerebral pial arterioles in the Control **a**, the Pre **b**, the Continuous **c**, and the Post **d** groups. Before global brain ischemia, Y-27632 produced pial arteriolar dilation (**b** and **c**). In the Control group, pial arterioles were constricted during the ischemia, and were dilated temporarily and then were constricted during the reperfusion period. In the Pre group, pial arterioles were not constricted during ischemia, and were temporarily dilated during the reperfusion period. In the Continuous group, pial arterioles were not constricted during the ischemia, and were dilated during the reperfusion period. In the Post group, pial arterioles were constricted during the ischemia, and were dilated during the reperfusion period. Ba = baseline, Y = after the application of Y-27632, I = initiation of global brain ischemia. * *P* < 0.05, versus baseline, † *P* < 0.05, versus the Control group, ‡ *P* < 0.05, versus the Pre group

**Fig. 2:**
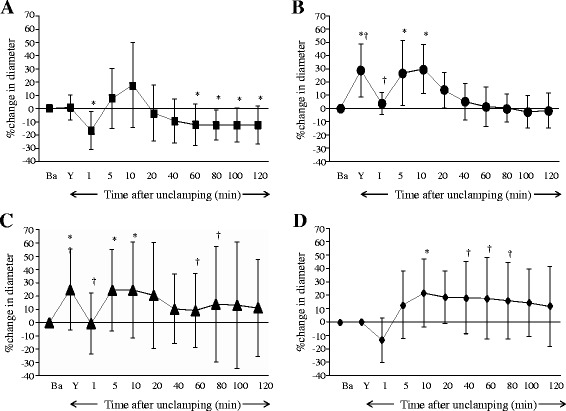
Percent changes in diameter in small (≤70 μm) cerebral pial arterioles in the Control **a**, the Pre **b**, the Continuous **c**, and the Post **d** groups. Before global brain ischemia, Y-27632 produced pial arteriolar dilation (**b** and **c**). In the Control group, pial arterioles were constricted during the ischemia, and were dilated temporarily and then were constricted during the reperfusion period. In the Pre group, pial arterioles were not constricted during ischemia, and were temporarily dilated during the reperfusion period. In the Continuous group, pial arterioles were not constricted during the ischemia, and were dilated during the reperfusion period. In the Post group, pial arterioles were constricted but not statistically significant during the ischemia, and were dilated during the reperfusion period. Ba = baseline, Y = after the application of Y-27632, I = initiation of global brain ischemia. * *P* < 0.05, versus baseline, † *P* < 0.05, versus the Control group

**Fig. 3:**
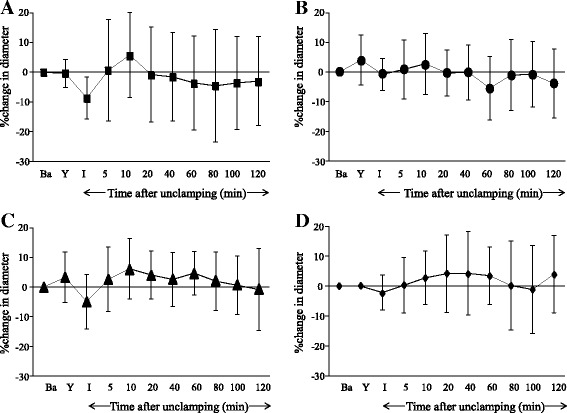
Percent changes in diameter in large (>70 μm) cerebral pial venules in the Control **a**, the Pre **b**, the Continuous **c**, and the Post **d** groups. Before global brain ischemia, Y-27632 did not produce pial venular dilation (**b** and **c**). Large venules remained unchanged during reperfusion in all groups. Ba = baseline, Y = after the application of Y-27632, I = initiation of global brain ischemia

**Fig. 4:**
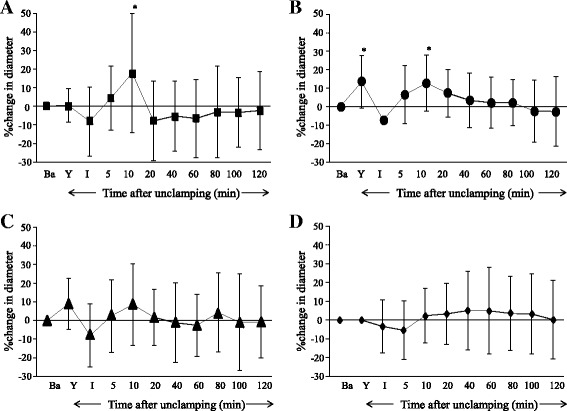
Percent changes in diameter in small (≤70 μm) cerebral pial venules in the Control **a**, the Pre **b**, the Continuous **c**, and the Post **d** groups. Before global brain ischemia, Y-27632 produced pial venular dilation **b**. During the ischemia, pial venules were slightly constricted but not statistically significant in all groups. During the reperfusion period, pial venules were dilated temporarily in the Control and the Pre groups but otherwise remained unchanged during reperfusion in all groups. Ba = baseline, Y = after the application of Y-27632, I = initiation of global brain ischemia. * *P* < 0.05, versus baseline

In the Pre and Continuous groups, topical application of Y-27632 at 10^−6^ mol∙L^−1^ produced large and small pial arteriolar and small venular dilation (Figs. 1b, c, 2b, c, and 4b), but did not significantly dilate venules (Figs. 3b, c, and 4c). During ischemia, large and small pial arterioles were constricted in the Control and the Post groups (Figs. 1a, d, and 2a), but remained largely unchanged compared to baseline in the Pre and the Continuous groups (Figs. 1b, c, 2b, and c). Pial large and small venules were slightly constricted but not statistically significant in all groups (Figs. 3 and 4).

During reperfusion in the Control group, significant constriction was observed in both large and small arterioles (Figs. 1a and 2a). In the Pre group, large arterioles were constricted, but not significantly (Fig. 1b). In contrast, pial arterioles were dilated in the Continuous and Post groups (Figs. 1c, d, 2c, and d). Small venular diameters increased temporarily after unclamping but otherwise remained largely unchanged during reperfusion in all groups (Figs. 3 and 4).

## Discussion

In this study, we observed that topical application of Y-27632 at 10^−6^ mol∙L^−1^ produced large and small pial arteriolar dilation, but did not induce venular dilation. Without Y-27632, pial arterioles were constricted at the ischemia, and were temporarily dilated and then were significantly constricted at the reperfusion period. These results concur with our previous studies [6, 7]. When Y-27632 was applied only before the global cerebral ischemia, pial arterioles were not constricted at the ischemia, and were temporarily dilated and then were constricted but not significantly at the reperfusion period. When Y-27632 was applied throughout the study period, pial arterioles were not constricted at the ischemia, and were dilated at the reperfusion period. When Y-27632 was applied from 10 min after the global brain to the end of the study, pial arterioles were constricted at the ischemia, and were dilated at the reperfusion period. Pial venules remained largely unchanged during ischemia and reperfusion period.

Generally, smooth muscle contraction is mediated by intracellular Ca^2+^ concentration. Elevated intracellular Ca^2+^ complexes with calmodulin and activates MLC. Activated myosin light chain kinase phosphorylates myosin light chain and phosphorylated myosin light chain induces smooth muscle contraction. The phosphorylation-state of myosin light chain depends on the balance of myosin light chain kinase activity and myosin light chain phosphatase activity. Specifically, myosin light chain is dephosphorylated by activated myosin light chain phosphatase, with consequent smooth muscle relaxation.

Rho-kinase mediates relaxation and contraction of smooth muscle independently of intracellular Ca^2+^ concentration [14–16]. Activated Rho-kinase inactivates myosin light chain phosphatase. Myosin light chain is therefore phosphorylated persistently and smooth muscle constricts. We previously reported that Y-27632, a Rho-kinase inhibitor, dilates cerebral pial arterioles [6]. As would thus be expected, topical application of Y-27632 at 10^−6^ mol/L dilated both large and small pial arterioles. Y-27632 is reported to induce concentration-dependent reduction of myogenic tone in the rabbit facial vein [17]. However, we did not observe similar dilation of pial venules that is consistent with our previous experience [6].

After brain ischemia of 15 min, pial arterioles and venules dilated in the control group; however, the arterioles thereafter constricted which is consistent with our experience in rabbits [7] and previous reports in mongolian gerbils [13]. Cerebral blood flow, which was measured by Laser Doppler flowmetry, and pial vascular diameter, which was measured with cranial window, are reported to change similarly [13]. Large intracranial and extracerebral vessels are a major site of regulating cerebral blood flow [18]. Therefore, vessels within the brain should respond similarly to pial vessels. Arteriolar constriction is associated with reduced capillary flow [13], and hypoperfusion after brain ischemia appears to contribute to development of cerebral edema [19] Additionally, Rho-kinase has been reported to be activated in the endothelial cells and contributes to microcirculatory disturbances in cerebral ischemia [9]. Rho-kinase may thus be a therapeutic target for ischemic stroke [4].

Pial arteriolar responses in the pre-application group were similar to those in the control group. In the Pre group, artificial cerebrospinal fluid was infused followed by the Y-27632 application. In the cranial window technique, drug's effect on pial arterioles is attenuated several minutes after the discontinuation of drug administration. It is therefore unsurprising that application of Y-27632 restricted to before ischemia did not produce vasodilation during reperfusion. In contrast, continuous application of Y-27632 before and during ischemia-reperfusion produced pial arteriolar dilation before and after ischemia-reperfusion. Consistent with this observation, Rho-kinase inhibition acutely augments blood flow in focal cerebral ischemia [20] and fasudil is antivasospastic in the context of subarachnoid hemorrhage [10].

In most clinical situations, brain ischemia cannot be reliably predicted. It is thus of considerable interest to consider the effects of Y-27632 when applied after onset of brain ischemia. We assumed that post-ischemic application of Y-27632 would produce less pial arteriolar dilation than application starting before ischemia. However, the amount of arteriolar dilation was similar under pre-ischemic and post-ischemic conditions, suggesting that it may be useful to give Y-27632 even after cerebral ischemia is identified. If we wish to leap to a potential therapeutic use of Y-27632, we should consider to administer it intravenously rather than topically, which would not be a reasonable route for clinical therapy.

Propofol is known to decrease the brain metabolism that is coupled with decreases in cerebral blood flow, though it has no pial vascular effects [21]. In the present study and our previous study [7], pial arterioles were dilated temporarily and were constricted continuously during the reperfusion period under propofol anesthesia. It is possible that propofol exaggerated the vasoconstriction during reperfusion. We also showed in the previous study that pial arterioles were dilated temporarily and were not constricted during the reperfusion period under sevoflurane anesthesia [7]. Sevoflurane is reported to produce cerebral vasodilation via activation of adenosine triphosphate-sensitive K^+^ channels [22]. Y-27632 and sevoflurane dilate pial vessels through different mechanisms. If we would have used sevoflurane in this study, Y-27632 may have exerted further dilating effects on pial arterioles.

In the present study, the group that received continuous application of Y-27632 had a reduced glucose response to global brain ischemia. It has been reported that Y-27632 enhanced glucose-stimulated insulin secretion [23, 24]. We did not measure the quantity of Y-27632 absorbed from the pial surface, when it was applied within the cranial window. However, some quantity of Y-27632 should have been absorbed from the pial surface to systemic circulation and induced glucose-stimulated insulin secretion at the global brain ischemia.

A limitation of our study is that cerebral blood flow was not measured. Nevertheless, laminar flow is proportional to 4th power of the vessel radius (Poiseuille's law), suggesting that the observed arterioloar dilation (i.e., 20–30% during reperfusion) markedly increased flow, perhaps by 2.0–2.9-fold. We did not include any histological analyses which would have characterized the presumed benefit on vasodilation on tissue. And finally, results in rabbits may extrapolate poorly to humans.

## Conclusions

Y-27632 dilated cerebral pial arterioles, but not dilate pial venules before ischemia and reperfusion preriod. Application of Y-27632 restricted to the pre-ischemic period did not prevent subsequent arteriolar constriction induced by ischemia-reperfusion. Both continuous administration and post-ischemic application of Y-27632 counteracted persistent cerebral pial arteriolar constriction during the reperfusion period. Continuous application of Y-27632 may thus be useful regarding the production of cerebral vasodilation after global brain ischemia, but also appears beneficial when it is applied after onset of ischemia.

## Abbreviations

aCSF: Artificial cerebrospinal fluid
ANOVA: Analysis of variance
ETCO_2_: End-tidal carbon dioxide tension
HR: Heart rate
MAP: Mean arterial blood pressure
MLC: Myosin light chain
PaCO_2_: Arterial carbon dioxide tension

## References

[1] Eto M, Barandiér C, Rathgeb L, Kozai T, Joch H, Yang Z (2001). Thrombin suppresses endothelial nitric oxide synthase and upregulates endothelin-converting enzyme-1 expression by distinct pathways: role of Rho/ROCK and mitogen-activated protein kinase. Circ Res.

[2] Li M, Huang Y, Ma AA, Lin E, Diamond MI (2009). Y-27632 improves rotarod performance and reduces huntingtin levels in R6/2 mice. Neurobiol Dis.

[3] Guan R, Xu X, Chen M, Hu H, Ge H, Wen S (2013). Advances in the studies of roles of Rho/Rho-kinase in diseases and the development of its inhibitors. Eur J Med Chem.

[4] Rikitake Y, Kim HH, Huang Z, Seto M, Yano K, Asano T (2005). Inhibition of Rho kinase (ROCK) leads to increased cerebral blood flow and stroke protection. Stroke.

[5] Chrissobolis S, Budzyn K, Marley PD, Sobey CG (2004). Evidence that estrogen suppresses rho-kinase function in the cerebral circulation in vivo. Stroke.

[6] Kotoda M, Ishiyama T, Shintani N, Matsukawa T (2015). Direct effects of Rho-kinase inhibitor on pial microvessels in rabbits. J Anesth.

[7] Ishiyama T, Shibuya K, Ichikawa M, Masamune T, Kiuchi R, Sessler DI (2010). Cerebral pial vascular changes under propofol or sevoflurane anesthesia during global cerebral ischemia and reperfusion in rabbits. J Neurosurg Anesthesiol.

[8] Nakano T, Okamoto H (2009). Dexmedetomidine-induced cerebral hypoperfusion exacerbates ischemic brain injury in rats. J Anesth.

[9] Yagita Y, Kitagawa K, Sasaki T, Terasaki Y, Todo K, Omura-Matsuoka E (2007). Rho-kinase activation in endothelial cells contributes to expansion of infarction after focal cerebral ischemia. J Neurosci Res.

[10] Satoh S, Takayasu M, Kawasaki K, Ikegaki I, Hitomi A, Yano K (2012). Antivasospastic effects of hydroxyfasudil, a Rho-kinase inhibitor, after subarachnoid hemorrhage. J Pharmacol Sci.

[11] Marchal G, Young AR, Baron JC (1999). Early postischemic hyperperfusion: pathophysiologic insights from positron emission tomography. J Cereb Blood Flow Metab.

[12] Martín-Cancho MF, Lima JR, Luis L, Crisostomo V, Carrasco-Jimenez MS, Uson-Gargallo J (2006). Relationship of bispectral index values, haemodynamic changes and recovery times during sevoflurane or propofol anaesthesia in rabbits. Lab Anim.

[13] Hauck EF, Apostel S, Hoffmann JF, Heimann A, Kempski O (2004). Capillary flow and diameter changes during reperfusion after global cerebral ischemia studied by intravital video microscopy. J Cereb Blood Flow Metab.

[14] Yoneda H, Shirao S, Nakagawara J, Ogasawara K, Tominaga T, Suzuki M (2014). A prospective, multicenter, randomized study of the efficacy of eicosapentaenoic acid for cerebral vasospasm: the EVAS study. World Neurosurg.

[15] Swärd K, Mita M, Wilson DP, Deng JT, Susnjar M, Walsh MP (2003). The role of RhoA and Rho-associated kinase in vascular smooth muscle contraction. Curr Hypertens Rep.

[16] Tani E (2002). Molecular mechanisms involved in development of cerebral vasospasm. Neurosurg Focus.

[17] Dubroca C, You D, Lévy BI, Loufrani L, Henrion D (2005). Involvement of RhoA/Rho kinase pathway in myogenic tone in the rabbit facial vein. Hypertension.

[18] Faraci FM, Heistad DD (1990). Regulation of large cerebral arteries and cerebral microvascular pressure. Circ Res.

[19] Hosomi N, Ohyama H, Ichihara S, Takahashi T, Naya T, Kohno M (2007). Relation of postischemic delayed hypoperfusion and cerebral edema after transient forebrain ischemia. J Stroke Cerebrovasc Dis.

[20] Shin HK, Salomone S, Potts EM, Lee SW, Millican E, Noma K (2007). Rho-kinase inhibition acutely augments blood flow in focal cerebral ischemia via endothelial mechanisms. J Cereb Blood Flow Metab.

[21] Shibuya K, Ishiyama T, Ichikawa M, Sato H, Okuyama K, Sessler DI (2009). The direct effects of propofol on pial microvessels in rabbits. J Neurosurg Anesthesiol.

[22] Iida H, Ohata H, Iida M, Watanabe Y, Dohi S (1998). Isoflurane and sevoflurane induce vasodilation of cerebral vessels via ATP-sensitive K^+^ channel activation. Anesthesiology.

[23] Hammar E, Tomas A, Bosco D, Halban PA (2009). Role of the Rho-ROCK (Rho-associated kinase) signaling pathway in the regulation of pancreatic beta-cell function. Endocrinology.

[24] Kong X, Yan D, Sun J, Wu X, Mulder H, Hua X (2014). Glucagon-like peptide 1 stimulates insulin secretion via inhibiting RhoA/ROCK signaling and disassembling glucotoxicity-induced stress fibers. Endocrinology.

